# Genomic signatures for predicting the zoonotic potential of novel viruses

**DOI:** 10.1371/journal.pbio.3001403

**Published:** 2021-09-29

**Authors:** Jason T. Ladner

**Affiliations:** The Pathogen and Microbiome Institute, Northern Arizona University, Flagstaff, Arizona, United States of America

## Abstract

Powered by metagenomics, viral discovery is outpacing our capacity for the downstream characterization needed to fully assess zoonotic potential. This Primer explores the implications of a PLOS Biology study which uses machine learning to prioritize novel viruses based on genomic signatures alone.

## Teaser

Powered by metagenomics, viral discovery is outpacing our capacity for the downstream characterization needed to fully assess zoonotic potential. A study published in PLOS Biology uses machine learning to prioritize novel viruses based only on genomic signatures.

**Fig 1:**
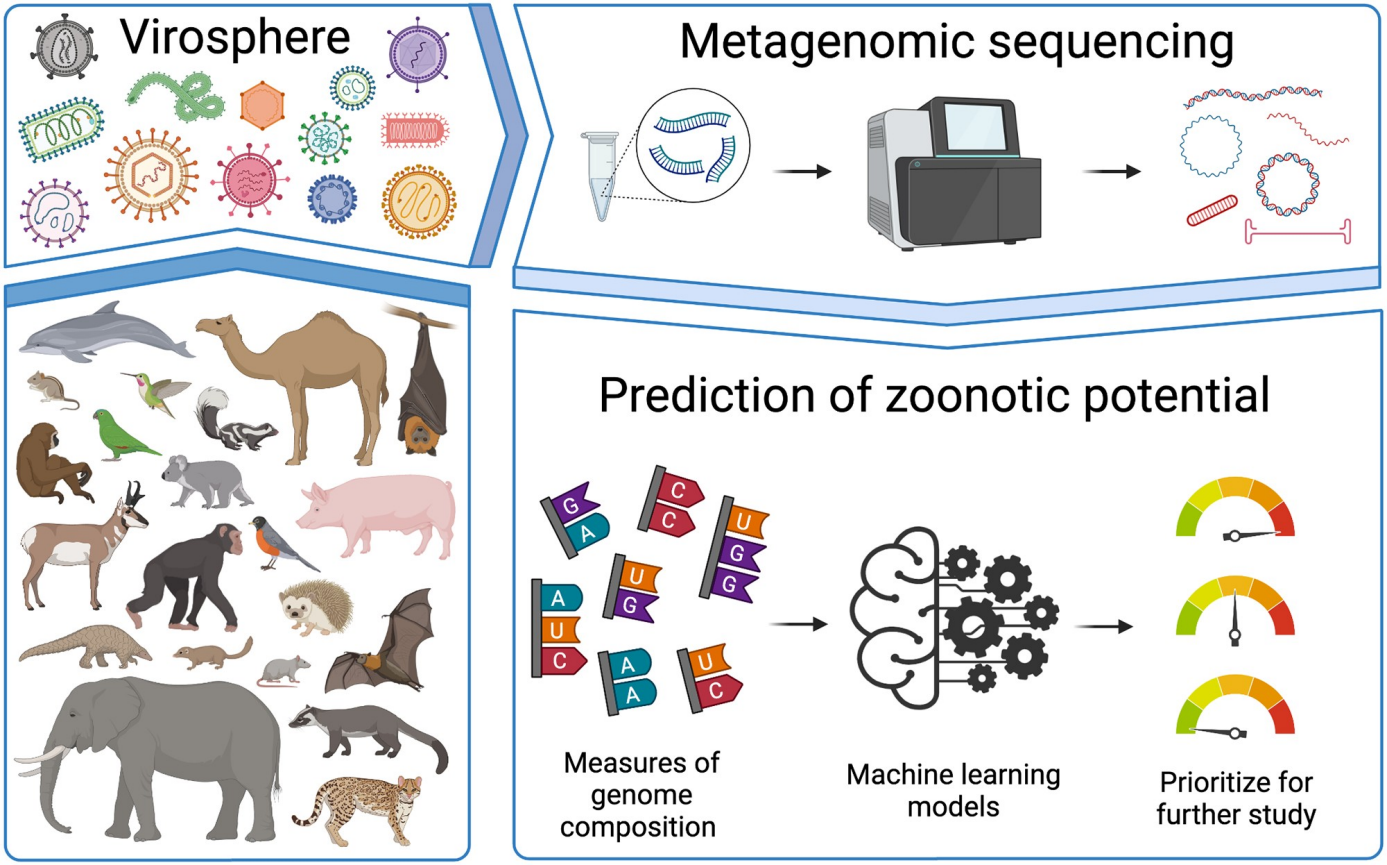
Predicting zoonotic potential using genomic signatures. Characterizing the diversity of viruses infecting non-human animals (i.e., the animal ‘virosphere’) is a critical component of pandemic prevention. However, current approaches for in-depth characterization cannot keep pace with the rate of sequence-based viral discovery driven by metagenomics. In this issue, Mollentze and colleagues present machine learning models that can prioritize novel viruses for follow-up studies based only on genomic signatures of zoonotic potential. Figure was created with BioRender.com.

The ongoing coronavirus disease 2019 (COVID-19) pandemic has provided a stark example of our vulnerability to emerging infectious diseases caused by viruses. Notably, COVID-19 is the result of human infection with a virus (SARS-CoV-2) that had never been observed prior to the start of the pandemic. This highlights a critical challenge for the prevention of future pandemics: how can we best prepare for a fight against an unknown opponent? Given the complexities of viral emergence, precise predictions are unrealistic. However, even though we cannot predict exactly which virus will emerge next, we can, and should, work to better understand the field of contenders, and in this issue, Mollentze and colleagues describe a new tool for doing just this. Using machine learning models, they present an approach for prioritizing novel viruses for further characterization based solely on compositional signatures present within viral genomes (Fig 1) [1].

In very general terms, we know from where novel viruses are likely to emerge. Most “new” viruses for humans are not actually new at all. Rather, they are viruses that have been infecting other animals and have only recently crossed the species boundary to infect humans. Therefore, if we want to be prepared for the next virus that will emerge in humans, we need to broadly characterize the viruses currently infecting other animals. This, however, is much easier said than done due to the immense diversity of viruses currently infecting animals, the vast majority of which remain completely uncharacterized. Even if we were to only consider viruses that infect mammals, our best estimates suggest that there are 10,000s–100,000s of undiscovered viruses [2].

High-throughput metagenomic sequencing has revolutionized the process and pace of virus discovery by facilitating agnostic and extremely deep characterization of DNA/RNA from clinical and environmental samples [2,3]. However, even if we succeed in using this approach to fully describe the diversity of viruses in animals (which remains an enormous task), this will still be the tip of the iceberg in terms of understanding the potential for these viruses to infect humans (i.e., zoonotic potential). This is because metagenomics only generates viral genome sequences, and the number of expected new viruses will make comprehensive characterization by more labour- and cost-intensive approaches (e.g., cell-culture and animal model studies) prohibitive. Therefore, we need sequence-based methods for prioritizing these new viruses for follow-up studies.

With this need in mind, Mollentze and colleagues developed machine learning models to predict the zoonotic potential of novel viruses based only on genomic signatures, which can be ascertained using metagenomic sequencing data. These signatures include 146 measures of viral genome composition bias (“viral features”). For example, the relative frequency of each codon, amino acid and dinucleotide. These viral features were also compared to the same metrics calculated for human RNA transcripts, thus providing measures of compositional similarity between human and viral sequences (“similarity features”). In contrast, traditional approaches have been based on overall measures of relatedness (e.g., taxonomy and genetic divergence) between novel viruses and viruses already known to infect humans.

Using sequences for 861 known virus species (from 36 families), Mollentze and colleagues show that their genome composition-based models for predicting zoonotic potential significantly outperform models based on measures of relatedness. This is true for both the viral and similarity features alone, and model performance increases further when these two feature sets are combined. In fact, their combined model predicted high or very high zoonotic potential for >70% of the viruses known to infect humans. This includes both zoonotic viruses and viruses maintained predominantly within the human population, and a very similar level of performance was observed for a set of 113 human-derived viruses that were not included in the initial training set.

Mollentze and colleagues also sought to understand why their models outperformed those based on relatedness, and what they found was that not only could their measures of genome composition broadly recapitulate evolutionary relationships, they were also able to capture generalizable features that may increase the likelihood for human infection. A great example of this comes from the Anelloviridae, which is a family of ssDNA viruses that was not represented in their initial training set. Their model assigned high or very high zoonotic potential to 39/45 (86.6%) of the human-associated anellovirus species they tested. Given the complete absence of viruses from this family during model training, these predictions must be based on features shared across human-infecting viruses from different families. At this point, Mollentze and colleagues can only speculate about the mechanisms underlying these generalizable features, but this is an area ripe for future experimentation, and by studying these signatures, we may be able to shed new light on the complex interactions that occur between virus and host.

In the coming years, the pace of virus discovery is likely to further accelerate. Therefore, sequence-based prioritization approaches, like that presented by Mollentze and colleagues, will be crucial, and the performance of these approaches is likely to improve as our understanding of the virosphere grows. However, it would be unrealistic to expect genomic analysis to solve all the challenges associated with comprehensive characterization of zoonotic viruses. Even using one of the lower estimates of virus diversity in mammals (40,000 total, 10,000 zoonotic) [4], the model described by Mollentze and colleagues would be expected to flag >3000 mammalian viruses as very high priorities and ~13,000 more as high priorities for further characterization. Therefore, we also need complementary, non-genomic triage approaches, like prioritizing viruses commonly encountered at the human-animal interface [2,5], and we need to improve our capacity for downstream characterization of novel viruses through the development and implementation of high-throughput and highly-multiplexed assays. For example, recent developments in serology now allow antibody reactivity against 100s-1000s of viruses to be assessed using a single assay [6,7]. In other words, models like those described in this issue by Mollentze and colleagues are welcome additions to our toolkit for pandemic prevention, but we still have a lot of work ahead of us.
